# A New Method of Airflow Velocity Measurement by UAV Flight Parameters Analysis for Underground Mine Ventilation

**DOI:** 10.3390/s25175300

**Published:** 2025-08-26

**Authors:** Adam Wróblewski, Aleksandra Banasiewicz, Pavlo Krot, Paweł Trybała, Radosław Zimroz, Andrii Zinchenko

**Affiliations:** 1 Faculty of Geoengineering, Mining and Geology, Wrocław University of Science and Technology, Na Grobli 15, 50-421 Wrocław, Poland; adam.wroblewski@pwr.edu.pl (A.W.); aleksandra.banasiewicz@pwr.edu.pl (A.B.); radoslaw.zimroz@pwr.edu.pl (R.Z.); 2 3D Optical Metrology Unit, Bruno Kessler Foundation (3DOM-FBK), Via Sommarive 18, 38122 Trento, Italy; ptrybala@fbk.eu; 3 Institute of Transport Systems and Technologies, National Academy of Sciences of Ukraine, Pissarzhevsky 5, 49005 Dnipro, Ukraine; zina@dsu.dp.ua; 4 Faculty of Informatics and Information Technologies, Slovak University of Technology, Ilkovičova 2, Bratislava 4, 842 16 Bratislava, Slovakia

**Keywords:** underground mine ventilation, Unmanned Aerial Vehicle (UAV), airflow velocity measurement, IMU sensor, drone flight parameters analysis

## Abstract

The idea of this research is to develop a method of airflow velocity measurement in underground mines having a network of long-distance crossing tunnels, where inspections of the ventilation system are required. Currently, this time-consuming procedure is conducted manually, but it has great importance when the mine configuration is subjected to changes. The method is based on the measurements of UAV trajectory deviation when it crosses the lateral air streams while moving along the tunnel. The signals of the gyroscope from the Inertial Measurement Unit (IMU) are used as indicators. The calibration of the proposed method has been conducted in laboratory conditions similar to real conditions. The minimal sensitivity of 0.3 m/s required by regulations is achievable for small drones, and the error is less than 5%. The maximum measured airflow velocity depends on the UAV model and its stabilization system. Recommendations are formulated for method implementation in practice.

## 1. Introduction

The dynamic development of modern technologies in the mining industry is evident. More and more often, solutions based on unmanned inspection and control of the working environment, existing hazards, or the functioning of machines and devices are used. The credibility of the use of Unmanned Ground Vehicles (UGVs) and Unmanned Aerial Vehicles (UAVs) in difficult conditions of underground mines has been proven in many studies. The implementation of robotic solutions can be divided into several main groups. Robotics for inspection applications is shown in [1,2,3], rescue missions with the use of robotics are described in [4,5], robotics for the diagnosis of technological equipment is presented in [6], and auxiliary technological processes are shown in [7,8,9].

In the mining applications mentioned above, UAVs are of great importance due to the variety of devices and sensors they can be equipped with [10], as well as their high mobility and relatively small dimensions. Additionally, a great advance is the ability to operate in conditions of poor-quality roads, where ground vehicles may encounter certain difficulties. Examples of drone use in underground mining can be found in [10,11,12,13,14,15].

Another application for drones in underground mines proposed by the authors is air velocity measurements. In underground mining works, air parameter monitoring is one of the key procedures that enables safe operation for the crew. For managing aerological hazards that occur in the mine, for example, thermal or gas hazards, it is crucial to measure airspeed values based on which a ventilation adequacy assessment can be performed. The minimum air velocity is used as a design parameter in ventilation engineering planning to provide the conditions required by the regulations and to keep miners safe. Thus, air velocity measurements in each active underground work are essential [16].

Before the invention of the vane anemometer, based on which airspeed measurements are mainly conducted, the only practicable method for measuring airflow rates in underground mines was dust or smoke observation. Although this method is practically displaced by measurements with the use of anemometers, it is still practiced sometimes for air movement or direction estimation [17].

Since the methods mentioned above have some imperfections, the attention of many scientists is focused on improving them. In [18], a new concept for the implementation of a three-axis ultrasound anemometer is presented. This idea allows for a more accurate determination of airflow by conducting measurements closer to the walls, which is difficult to obtain with a traditional vane anemometer. The concepts of real-time air velocity monitoring systems are presented in [16,19,20].

Continuous monitoring of airflow velocity in mining excavations allows for the elimination or at least reduction in the presence of people from ventilation surveys working under dangerous conditions that prevail in mines. However, implementing this solution and ensuring its practical operation in the conditions of an underground mine is not an easy task. Sensors for data collection must be placed in crucial places from a ventilation point of view. Usually, they are attached to the excavation roof on outriggers of a certain length.

One should remember that mining excavations are the workplace for various types of heavy equipment, such as loaders, dump trucks, etc. Installing sensors on the roof of the workings, especially in conditions of low heights, in the order of 1.5–2 m, as in the case of Polish copper ore mines, may limit the mobility of machines crucial for the mining process. For this reason, such a solution may thus have limited applicability.

Promising results for air velocity measurements have been obtained by drone application. Their high mobility and relatively small size enable trouble-free cooperation with other machines operating in the mine. The variety of sensors with which these platforms can be equipped allows one to monitor such key air parameters as velocity. Another important advantage of using drones to perform measurements is the reduction in the presence of people in dangerous conditions (dust, high temperature, humidity, and exhaust gases) due to the possibility of remote control of these devices from safe places.

The innovation in this paper is a new method for air velocity measurements in underground mining excavation using a small UAV. The proposed method is based on the assumption that the main disturbance and deviation of the drone trajectory are caused by the lateral airflow while crossing the open spaces of numerous rooms between the pillars. This method is universal and also applicable to any other underground environment. The developed method of airflow velocity measurements is mostly suitable in underground mines with room and pillar exploitation systems when a network of long-distance crossed tunnels needs scheduled inspections of ventilation performance. The automatic procedure of data collection over long distances of underground tunnels (100–200 m for a single measurement and kilometers for the complete inspection mission) greatly improves the reliability of the results. In addition, digital data are provided for further CFD analysis and modeling of ventilation systems. The airflow velocity measurement accuracy achieved (maximum error of less than 5% and average error of about 2% compared with the anemometer) is allowable for practical applications.

The paper is organized into the following sections: a detailed overview of airflow velocity measurement methods in underground mines is given in Section 2, the experiments conducted in a calibration station with an anemometer and drone measurements in Section 3, measurement data analysis and CFD simulations in Section 4, discussion of results in Section 5, and conclusions in Section 6.

## 2. State of the Art

### 2.1. Methodology of Airflow Measurements in the Underground Mines

Airflow velocity measurements are made in underground works to determine the speed of free airflow and to allow the calculation of volume flow or air quantity [21]. The flow velocity is highest in the central part of the excavation and gradually decreases to zero towards its walls. Since mining excavations are usually irregular and very variable in shape, as well as the presence of various types of objects, sometimes moving, the field of airflow velocity is also irregular [22]. Therefore, determining the volume flow based on air velocity measurements at one point in the excavation would be unreliable. Ventilation services in mines usually use a method called a traverse.

**Figure 1:**
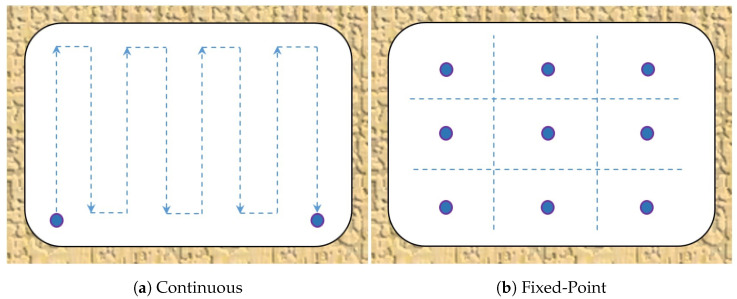
Traverse methods (developed based on [24]).

Traverse is a method for air velocity measurements in an airway, in which the instrument, usually an anemometer, is moved across the cross section of the airway or part of it [23]. The traverse could be fixed-point (Figure 1b) or continuous (Figure 1a) and is applicable for steady flow. In the point traverse method, the cross section of an airway is divided by the use of a string or wire into particle fields *n* of approximately the same area. At the middle point of each particle field, the air velocity $v_{i}$ is measured with the anemometer, showing an instantaneous speed. The average velocity of air in the whole cross section of excavation $v_{av}$ is determined as a weighted average of the measured values in each field $F_{i}$ by the following equation [22]:

(1) $$v_{av}=\frac{1}{F}\sum_{i=1}^{n}v_{i}F_{i}$$

where *n*—number of particle fields, $F_{i}$—area of *i* particle field ($m^{2}$), $v_{i}$—air velocity in *i* particle field (m/s), and *F*—area of excavation ($m^{2}$).

The airflow volume $\dot{V}$ is determined by the following equation [22]:

(2) $$\dot{V}=v_{av}F=\sum_{i=1}^{n}v_{i}F_{i}$$

Another method of determining air velocity in underground mines uses devices called velometers. A velometer is an instrument capable of making instantaneous measurements of airflow precisely at specific points with the probe. Such devices are used more for checking the velocity at a fixed point than for tediously traversing the cross section of the airway [23].

When the air velocity underground is too small to be detected by anemometers, the timed smoke cloud method is applied. In a smoke tube, smoke clouds are generated at a certain time. When the distance between the measurement and the travel time of the smoke cloud is known, the airflow velocity can be obtained by dividing the distance that the smoke cloud traveled by the travel time [23]. On the other hand, the value of the air velocity measured at a single point can be mathematically transformed to an average value flowing through the airway using the correction factor [21]. The method for determining the correction factors is shown in [24]. This method uses the ratios of the measured average air velocity, gained, e.g., by the traverse method, to the measured single-point air velocity.

### 2.2. Wind Velocity Measurements with UAVs

Different methods of airflow velocity (wind speed) measurement are known and realized in the following corresponding devices:

- Vane anemometers where propeller rotation is proportional to airflow velocity.
- Hot-wire probes based on the cooling effect of airflow on a heated wire resistance.
- Sonic or ultrasonic anemometers where the time for the sound to travel between the transducers depends on wind speed.
- Laser Doppler anemometers analyze the Doppler shift in laser light reflected from airborne particles.
- Laser Light Detection and Ranging (LIDAR) systems measure the reflection times from particles in the air.

Some of these compact digital devices or sensors can also be mounted on the UAVs for different tasks, e.g., air sampling [25] and other atmosphere measurements [26,27] including UGV platforms [28]. However, if the anemometer is embedded in the UGV, this restricts measurements to the ground boundary level. Otherwise, a special mast with a regulated height should be installed on the UGV to reach different levels of the tunnels, which greatly restricts their application in the underground conditions. In addition, severe disturbances, such as uneven roads and surface watering in underground mines, will cause inaccurate measurements due to long mast oscillations. Autonomous UGVs, due to their slow motion compared with UAVs, cause an inefficient use of personnel time during ventilation inspections in harsh conditions. The cost of wheeled or especially legged UGVs is much higher than that of any serial UAV model [29].

As mentioned in [30], the measurement of useful parameters that characterize the safety and flight performance of large UAVs is easy to obtain. Such vehicles can be equipped with numerous devices, such as vanes or multi-hole pitot-static tubes, since their size, weight, and power consumption are not an obstacle. Their proper calibration enables one to get information about wind velocity, angle of approach (AOA), or side-slip angle (SSA). The situation differs in the case of small UAVs, which, due to their size, cannot be equipped with such a variety of measurement devices. However, the possibility of estimating parameters of interest can be gained with the use of sensors, which are the basic equipment of every small UAV.

The Inertial Measurement Unit (IMU), which constitutes the basic equipment of small UAVs, provides information about its position, thus its orientation, gravitational force, and velocity (angular and linear). The standard IMU consists of a gyroscope, accelerometer, and magnetometer, mostly triaxial to obtain measurements on all three axes [31]. The output data of these devices are usually converted from analogue to digital form through an ADC, providing the data necessary to control the position of the UAV.

For on-surface flying UAVs, even moderate wind poses a significant challenge to navigation and in-flight stabilization. Many publications describe methodologies on the issue of airflow measurements online as the main disturbance factor of flight. In the work of Wenz et al. [32], the two most commonly used methods are mentioned to estimate airflow variables and aerodynamic coefficients: the Extended Kalman Filter, presented among others in [33], and the Unscented Kalman Filter, presented among others in [34]. Wenz et al. [35], but also other scientists [36], made some efforts to extend and improve the results gained by the application of these methods, taking advantage of a Global Navigation Satellite System (GNSS), an Inertial Measurement Unit (IMU), or even a digital camera. The approach to wind speed prediction accuracy based on attitude data derived from a high temporal frequency accelerometer from two different small UAVs and two machine learning (ML) algorithms is presented in [37]. Since the presence of wind is one of the most important factors affecting UAV trajectory drifts, the accurate determination of wind velocity enables drift reduction and, consequently, better stabilization and overall performance of a guidance system. The concept of wind velocity estimation, with the support of the above-mentioned standard sensor kit, was also born from the need for improvement of tracking pre-defined paths by the UAVs, as a part of an algorithm for its guidance. This idea is presented among others in [38].

However, remember that, due to the underground mine conditions, utilizing GNSS is not possible due to a lack of coverage [39]. For such conditions, only the utilization of the remaining standard UAV sensors, especially IMU sensors, seems to be the only means to estimate airflow velocities.

**Figure 2:**
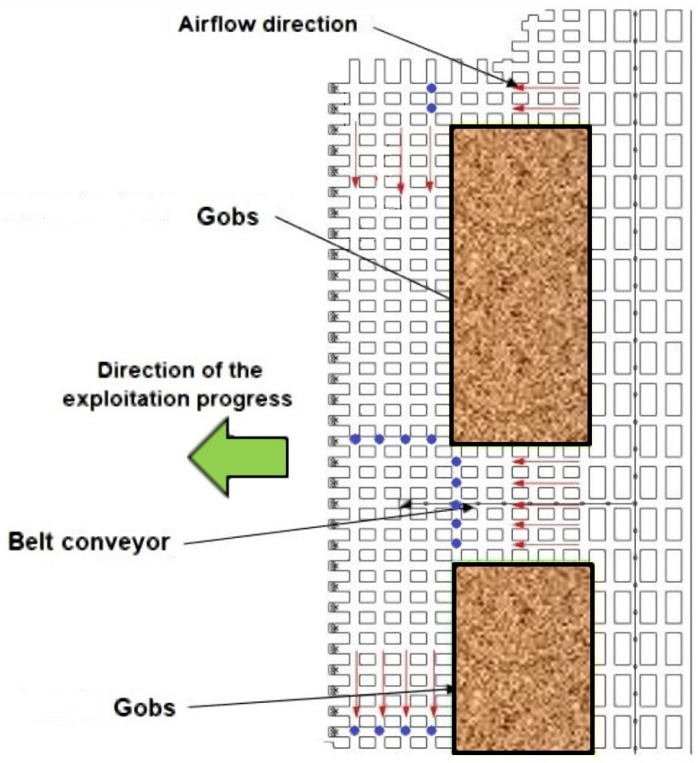
An example scheme of ventilation and exploitation works with a room and pillar system (developed based on [41]).

Due to geological and mining conditions, the most appropriate system for the exploitation of copper ore deposits in Poland is a room and pillar system. There are many types of this system used in the mines of KGHM Polska Miedź S.A. (Lubin, Poland), depending on the specific conditions in the given mining regions. Generally, the room and pillar system assumes cutting the deposit into suitable technological pillars, with the location of the longer axis perpendicular to the progress line of the mining front [40]. The exploited space is usually liquidated by cutting up the technological pillars into the residual pillars, thus enabling the convergence of the workings and creating the so-called gobs. This method of liquidation is called ceiling deflection. When it is necessary to prevent the lowering of the area above the mine, the exploited space is liquidated using a hydraulic backfill. An exemplary diagram of the exploitation and ventilation of work areas using a room and pillar system with a deflection of the roof and a movable closing pillar for the belt conveyor is presented in Figure 2.

The airflow directions are marked with red arrows. Air velocity measurements with the use of UAVs can be made in places of high variability of airflow, where it will be possible to disturb the flight trajectory, i.e., in places where airflows are combined or separated and where their flow directions change. For the ventilation conditions that prevail in mines using the room and pillar exploitation system, these will therefore be places where the UAV flight path will be perpendicular to the airflow directions marked with red arrows. The places in which measurements can be made are marked with blue dots.

## 3. The Experiment

To prove the concept of airflow measurement developed during ventilation inspection in underground mines, laboratory experiments were carried out at the facilities of the Faculty of Geoengineering, Mining, and Geology of Wroclaw University of Science and Technology.

### 3.1. Laboratory Tests on Anemometer Inclination Influence on the Air Velocity Measurements

**Figure 3:**
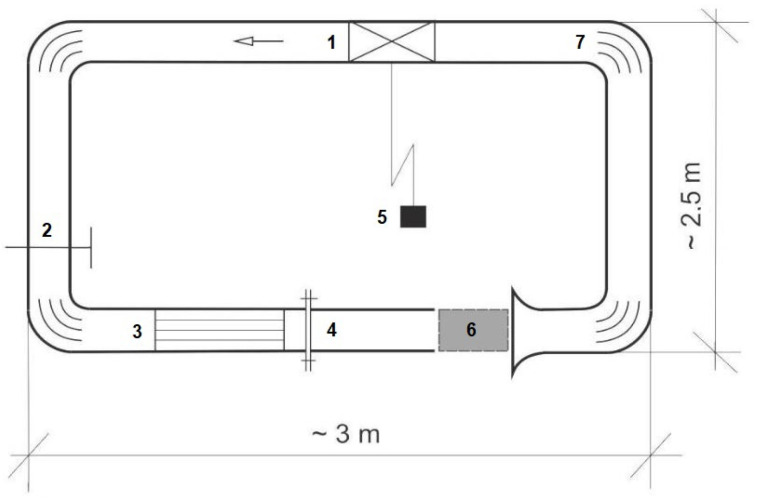
Anemometer calibration station: (1) duct fan, (2) shut-off gate valve with replaceable partition, (3) air-stream align element, (4) exchangeable section of the pipeline allowing changes of the length of the measuring area (0.12–0.2 m), (5) inverter for fan operation control, (6) measuring area, and (7) air-stream align element in the elbow.

**Figure 4:**
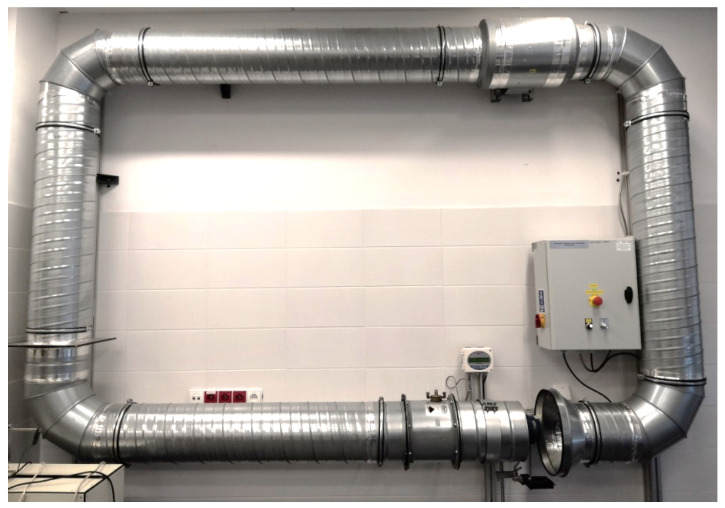
Anemometer calibration station.

First, the experiment was planned and carried out to quantitatively estimate the influence of the inclination of the anemometer axis toward the airflow on the measured values. The experiment was carried out on an anemometer calibration station (see Figure 3 and Figure 4). This is very important in both measurements in the mine and further laboratory experiments with UAVs.

**Figure 5:**
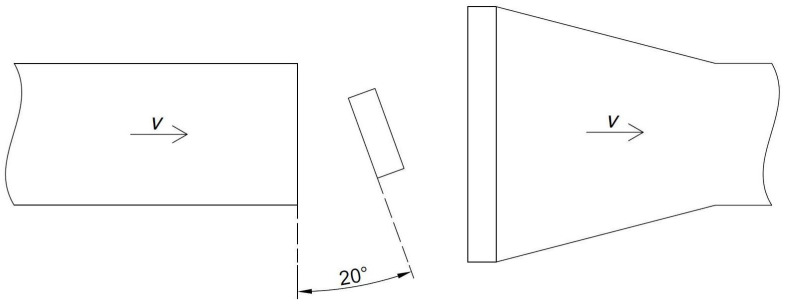
The anemometer position scheme to the air velocity vector in the pipeline (20°)—top view.

**Figure 6:**
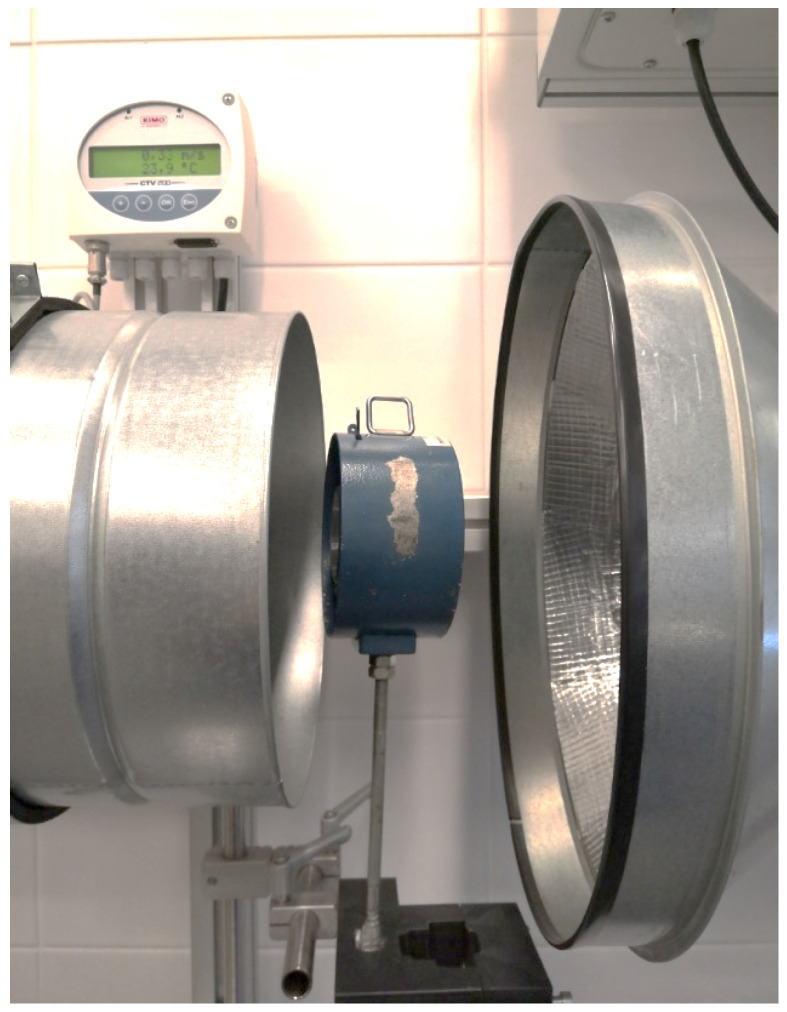
Anemometer setting in the calibration station.

Measurements were made for four different air velocities in the station pipeline (0.50, 3.40, 5.60, and 8.80 m/s) and three different angles of anemometer setup to airflow direction (0°, 10°, and 20°) (see Figure 5 and Figure 6).

The purpose of this experiment was to determine the influence of the inclination angle of the anemometer toward the velocity vector of the airflow on the velocity measurement. For each of the airflow velocities in the pipeline, velocity readings were taken on the anemometer at its parallel setting with the pipeline cross section, as well as when turned 10° and 20°. The results of this experiment are presented in Table 1.

The data in the table represent the air velocity values measured at the calibration station. During measurement on the laboratory stand, the fan parameters were set, the value of the speed in the pipeline outlet was read from the display, and then this speed was measured with the standard manual anemometer. The measurements were divided into two stages. In the first stage, the anemometer was positioned perpendicular to the air stream so that the outlet air flowed along the anemometer’s axis at the station. The next stage consisted of changing the angle of rotation of the anemometer by 10° and 20°. Measurements were taken at four different speeds, with the lowest value (0.3 m/s) corresponding to the minimum value of air that must flow through the excavation, while the highest value (7.4 m/s) was close to the maximum air speed in the excavation (8.0 m/s) [42].

When analyzing the measurement results at an angle of 0° (normal), it can be seen that the speed values measured by the anemometer differ from those read from the computer display of the station where different types of sensors are installed (electric). This difference increases as the airflow speed increases. The reason for these deviations is that an electrical sensor is installed in the tube while the anemometer is between the tubes, where air escapes through the pipeline connection slot. In addition, a slight error in the measurement, when determining the average speed, is better compensated for in the electronic version of the device.

Additionally, as can be seen from Table 1, the small decrease of air velocity with a rising angle from the measurement axis is caused by the non-axial incidence of airflow on the anemometer blades as well as the partial air braking by the anemometer housing, which constitutes a resistance in the event of inclination.

**Figure 7:**
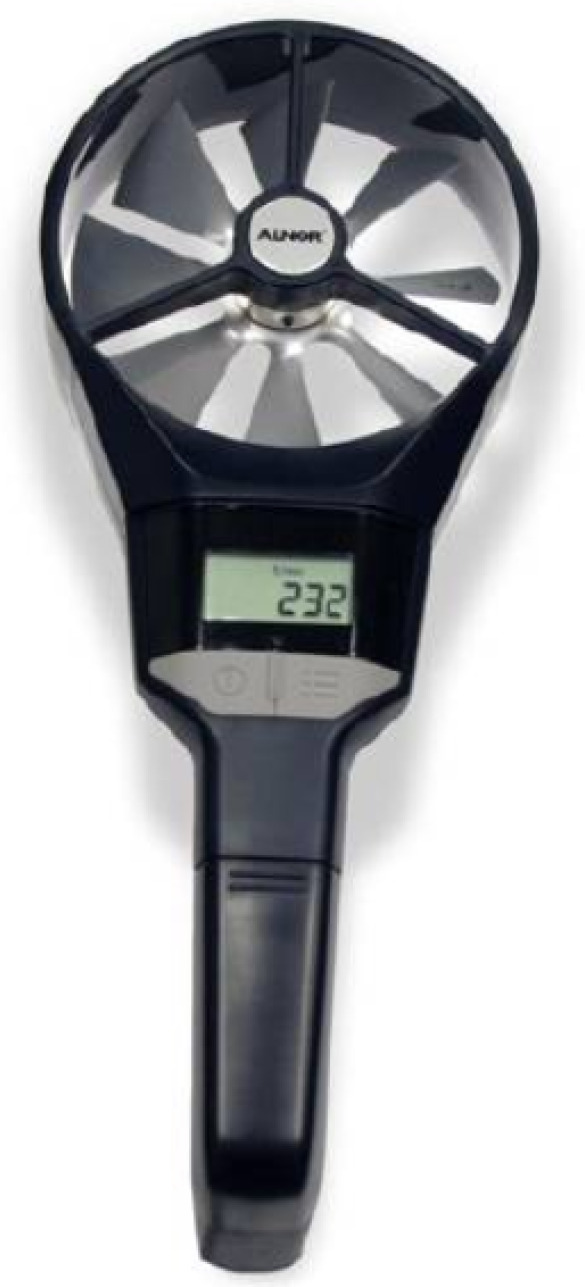
Rotating vane anemometer model LCA301 (TSI Airflow Instruments, Shanghai, China).

In general, the measurements by the manual anemometer are consistent for several levels of airflow and angles of inclination of the axis. Following the results of the experiments, the inclination of the anemometer within the visually distinctive range of angles can be neglected. Further experiments were conducted with a manual anemometer, LCA301 (TSI Airflow Instruments, Shanghai, China) (see Figure 7). This device has an accuracy of ±1.0% within a range of 0.25–30 m/s, which covers all values observable in practice.

### 3.2. Drone Measurement System

The proposed concept of the experiment assumes measuring the air speed in a point-like manner with a drone. The deviation is a result of the tilting of the UAV during measurement. It is influenced by airflow in the excavation or a blast of air from another excavation at the intersection of tunnels. During the experiment, a commercially available DJI Mavic Mini drone (DJI, Shenzhen, China) was used. This small quadcopter, weighing 249 g, using 4 pairs of light, 119-mm diameter propellers, was controlled manually. The reason why this device was chosen is that it represents well the wider class of Micro-Aerial Vehicles (MAVs), which are the most suitable type of unmanned vehicle to be used in an underground mine, due to their small size and fairly good battery life. Moreover, the drone’s relatively low weight allows for its distinct displacement in the presence of even low-speed side airflow, which can be recorded by the internal IMU sensor. Furthermore, since the experiment was carried out indoors, the propeller guard provided by the UAV manufacturer (DJI, Shenzhen, China) was mounted on the UAV to avoid damage if it collided with the walls.

**Figure 8:**
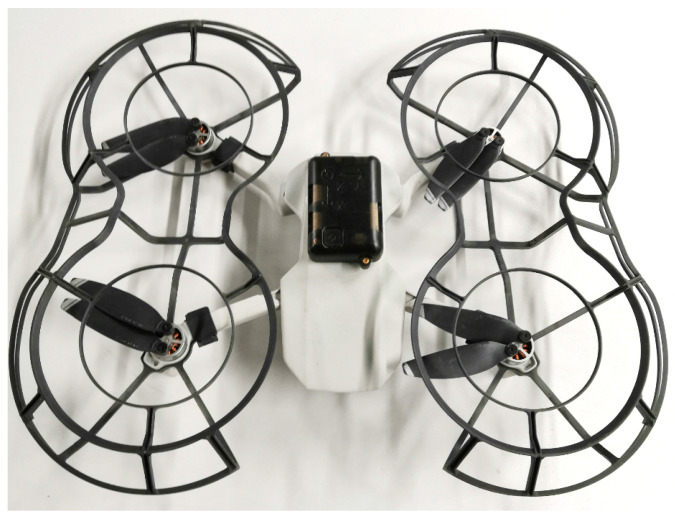
DJI Mavic Mini with additional IMU sensor.

Although the UAV used is factory-equipped with the IMU sensor, the data gathered by this device is limited to only 20 Hz. Thus, to investigate dynamical processes in more detail, an additional IMU sensor was attached to the UAV (Figure 8). The external module used is a commercially available NGIMU (New Generation IMU) from x-io Technologies (Bristol, UK), which is equipped, among other sensors, with a 3-axis accelerometer, gyroscope, and magnetometer. In addition, the system also has Wi-Fi data transmission and is supplied with software for real-time signal logging with a sampling frequency of 400 Hz (gyroscope, accelerometer). All data can also be stored on an internal SD card. Every signal is time-stamped; therefore, although some messages are lost during transmission, timestamps ensure the robustness of recording and further analysis.

The NGIMU module has a special internal function to calibrate the gyroscope (±2000°/s) and accelerometer (±16 g) when the device is in a stable position. Hence, zero bias values (offsets) are provided during experiments for both channels.

The minimal weight of this external module (10 g board and 36 g housing) and moderate price (GBP 310) allow installation on many comparatively low-cost UAVs for ventilation inspection.

**Figure 9:**
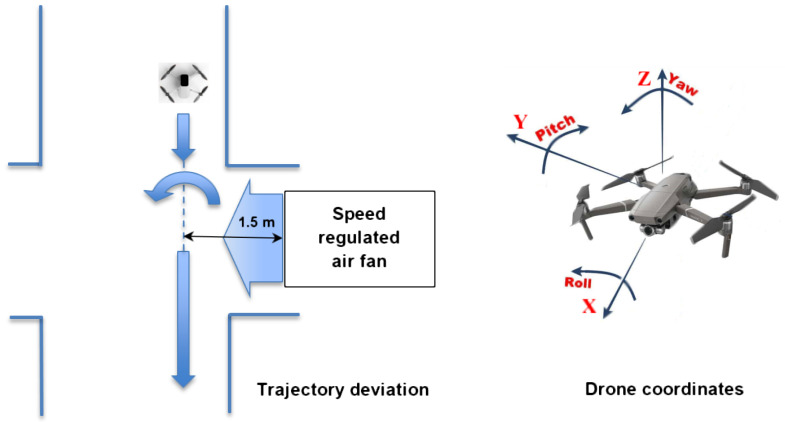
The plan of the experiment with coordinates of the UAV flying in the corridor and the trajectory deviated by the air fan installed in the side room.

A laboratory fan with five-stage rotation speed control was used to simulate airflow ranges to create the ventilation flow conditions of the underground mine. The fan was located in the corridor perpendicular to where the UAV operated. The plan of experiments and the standard spatial coordinates of the UAV are shown in Figure 9.

Before each experiment, the airflow velocity generated by the fan was measured using a manual anemometer in the corridor, at the UAV flight trajectory (about 1.5 m from the fan; see Figure 9). The airflow velocity values obtained this way were (1) 1.13, (2) 1.34, (3) 1.62, (4) 1.78, and (5) 2.40 m/s. The trajectory of the drone flying into the side airflow generated by the fan was disturbed and deviated, which was recorded by both internal and external IMU sensors.

**Figure 10:**
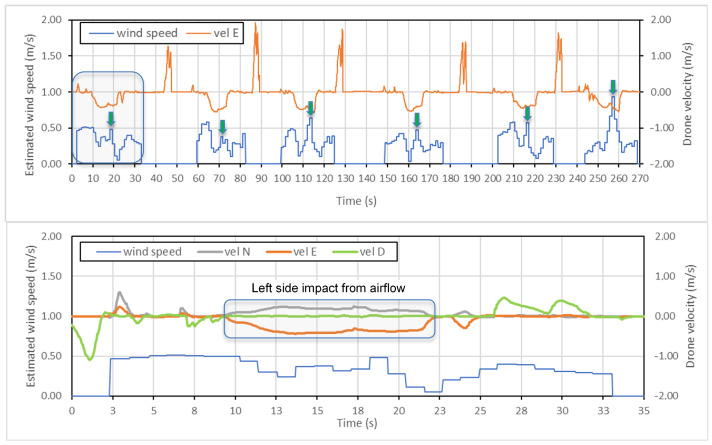
The estimated wind velocity and drone velocity signals from the internal IMU sensor (20 Hz) when measuring airflow velocity (1.13 m/s).

**Figure 11:**
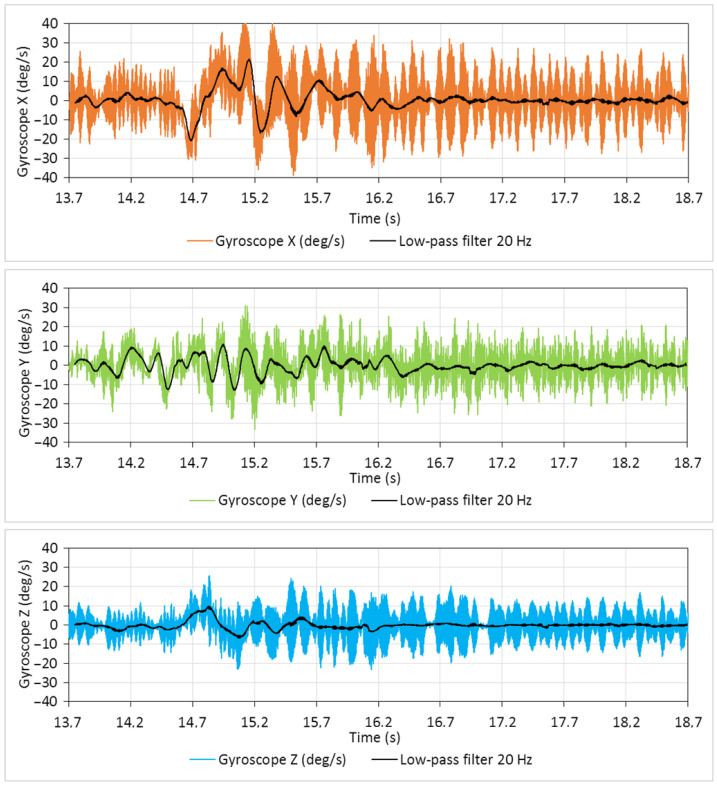
The original and low-pass filtered signals of gyroscope, roll (X), pitch (Y), and yaw (Z) angular rates from the external IMU sensor (400 Hz) when measuring airflow velocity (1.13 m/s).

The drone velocity was kept as constant as possible throughout every series of experiments. The stability of drone flight in experiments beyond the transient periods of the side airflow action can be estimated in Figure 10 (longitudinal velocity) and Figure 11 (rotational components). Additionally, since the gyroscope rotation signal is used instead of lateral acceleration as a parameter for airflow measurement, it is less sensitive to small deviations of the UAV’s longitudinal velocity.

## 4. Measurement Data Analysis

### 4.1. Internal IMU Sensor

The combined analysis is implemented for data available from the internal drone flight log and the external IMU module. First, the available standard signals for the estimated wind speed and drone velocity are analyzed, which are represented in Figure 10. The second graph shows the zoom of one measurement flight in which all components of drone velocity are present related to the north (N), east (E), and down (D) directions. The internal IMU sensor gives the velocity and some other parameters in the geodetic system of coordinates. The angle between the drone flight line and the north direction was 289°. Therefore, both the N and E components of the velocity change during the impact of the airflow from the fan.

Looking at the graphs and following the calculations, the estimated wind velocity values are not consistent with the value applied in this experiment (1.13 m/s). The peak values of estimated wind velocity shown in the first graph have large deviations, even with the one experiment, when laboratory conditions excluded any other external impacts on the drone, which may cause velocity deviations. Hence, some other parameters and signal processing methods are needed for wind velocity estimation, including data from the external IMU sensor.

### 4.2. External NGIMU Sensor

The original signal from a gyroscope is recorded with a sampling frequency of 400 Hz. Preliminary resampling is also implemented for the original signals since the sampling rate deviated in the 0.000–0.005 s range with a mean value of 0.0025. In parallel to raw data, the low-pass (20 Hz) filtered transient signals are shown in Figure 11.

Among other data recorded by the NGIMU module, the gyroscope signals appeared to be more sensitive to external disturbances related to side airflow changes. The side airflow from ventilation is a step-like disturbance, which causes the decaying oscillations of the drone body due to its PID regulators of the stabilization system. The duration of oscillations (about 1.0–1.5 s in our case) depends on the closed-loop regulators’ parameters tuning, while the maximum deviation depends on the disturbance amplitude (side flow velocity). The angular speed (deg/s) around the X (roll) axis is determined to be the most appropriate for measuring the airflow velocity. In particular, the value $X_{max}$ of the maximum deviation of the transient trajectory is taken for analysis. To correlate the parameter $X_{max}$ with the external airflow velocity, experiments were carried out in six series for each of the five airflow velocities.

### 4.3. CFD Simulation of Real Underground Tunnel

The CFD simulation of airflow in the tunnel was performed to analyze the restrictions and applicability of the proposed method. It is a proven method for ventilation analysis in underground mines [43,44]. The FEM model was created based on the 3D scans of the authors of full-size real tunnels by LIDAR in the underground mine [45,46]. In this study, the goal of the CFD simulation was to understand the possible effect of the UAV on the airflow velocity measured and its distribution in the tunnel.

**Figure 12:**
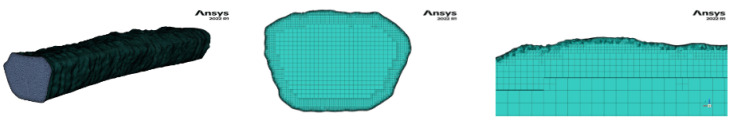
Tunnel space mesh in CFD simulation of airflow.

**Figure 13:**
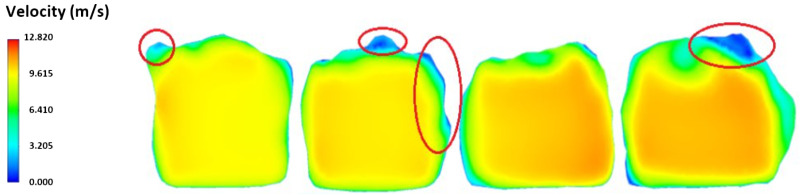
Results of CFD simulation of airflow in the tunnel with the low-velocity boundary regions (red circles).

The tunnel space mesh in the CFD simulation of airflow is shown in Figure 12, which has a higher density near the surface, since turbulent flows are expected there. The results of the CFD simulations are shown in Figure 13. Following these results obtained from the simulation, we can conclude that, in the central part of the section, where the main volume of air is flowing, the velocity is quite evenly distributed. Only the thin turbulence layer (up to 0.05–0.10 m) is observed along the perimeter near the wall surface. This distribution is valid for small deviations from the nominal tunnel profile. However, there are certain changes in the velocity of airflow along the tunnel. In the case of large unevenness, this turbulent layer is thicker and can reach 0.2–0.3 m depending on the roughness of the wall, increasing the resistance to ventilation [47].

**Figure 14:**
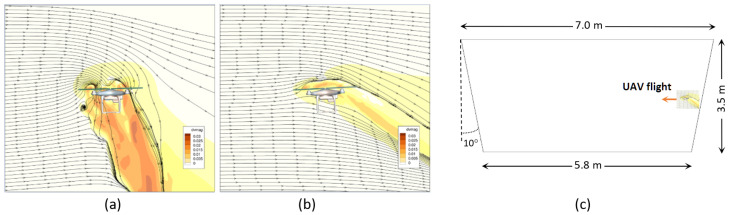
Velocity field perturbations and steady streamlines around a drone flying at (**a**) 2 m/s and (**b**) 5 m/s forward velocity [48]; (**c**) the nominal geometry of the tunnel section and scaled size of the drone with disturbed surrounding area for 5 m/s velocity.

Since the CFD simulation of UAVs is quite well represented in the literature, e.g., [48,49,50], we used the results of other authors to estimate the possible influence of the propeller-induced airflow of the drone itself on the measured airflow velocity. Figure 14 shows the results of the CFD simulation for the drone (DJI Phantom 3, (DJI, Shenzhen, China) with two horizontal velocity values of 2 m/s and 5 m/s taken from work [48].

From the results of the CFD simulations, we can conclude that the lower the horizontal velocity of the drone, the larger the area of disturbance in the surrounding air. However, this area by square is less than several drone body sizes for a 2 m/s velocity. This proportion is even lower for small drones, like the DJI Mini, which was used in experiments. However, a higher velocity will result in a smaller deviation of the drone caused by side airflow, which we intend to measure. Based on these simulation results, the horizontal velocity should be at least 2 m/s. To avoid the effects of the proximity of the ground, walls, and ceiling on the measurements, the minimum distance should be 0.5 m for such a small drone as the DJI Mini. This limitation also allows us to avoid turbulence near the ceiling and floor.

The square of the trapezoid tunnel section is about 22.4 $m^{2}$, and the square of the drone body with a disturbed area is about 0.5 $m^{2}$ or about 2% for its speed greater than 2 m/s. Hence, we can consider that the proposed method of airflow measurement with such a ratio of tunnel section and drone disturbed area can be considered non-invasive.

The laboratory tests should be related to real conditions in the underground mine, and appropriate geometric, kinematic, and dynamic similarity conditions must be met. Geometric similarity requires that the real object and the model object be geometrically similar. The size of the corridor is (W × H × L): 3.2 × 3.4 × 20 m, which is similar to an underground tunnel, the geometry of which is given in Figure 14.

Kinematic similarity requires similar velocity fields in the flow between the real mine and the corridor. This criterion is also satisfied, as the air and drone velocities were the same as those of the real mine. The main parameter, which can potentially create a difference (non-similarity) between laboratory tests and real mines, is the air temperature via its density and the Reynolds number, accordingly.

The dynamic similarity requires the similarity of the force fields that occur in the real and model objects. In many cases, it is not possible to obtain full similarity. The experimental tests are then carried out with partial similarity. This requires special corrections to consider the so-called scale effect when converting the results to the real object.

The room temperature in our tests was about 20 °C and is usually about 30–35 °C in the underground mine. For these temperature values, the kinematic air viscosity is 1.51 × $10^{-5}$ $m^{2}$/s and 1.65 × $10^{-5}$ $m^{2}$/s, respectively. The Reynolds number that describes the relation between inertial and viscous forces is as follows:

(3) $$Re=\frac{u\cdot L}{\nu },$$

where *u*—flow velocity (m/s), *L*—characteristic length (m), and ν—kinematic viscosity ($m^{2}$/s).

The characteristic length is as follows:

(4) $$L=\frac{4\cdot A}{p},$$

where *A*—section area ($m^{2}$) and *p*—section perimeter (m). The dimensionless Reynolds numbers are 2.18 × 10^5^ and 2.74 × 10^5^ for the corridor and tunnel, respectively. That is, both cases exhibit flow turbulence (Re>4000) of comparable magnitude.

One more dimensionless parameter, which determines the flow conditions at the outlet of the channel, is the Froude number: subcritical (Fr<1), critical (Fr=1), and supercritical (Fr>1). The Froude number is calculated by the following formula:

(5) $$Fr=\frac{u^{2}}{g\cdot H_{d}},$$

where *u*—flow velocity (m/s), *g*—acceleration due to gravity (m/$s^{2}$), and $H_{d}=A/p$—hydraulic mean depth equals the ratio of area to perimeter of the channel (m). In laboratory experiments for a maximum flow velocity of 2.40 ${m/s}^{2}$ and for mine conditions, these Froude numbers are 0.84 and 0.72, respectively. That is, both are below the critical value (Fr = 1) that corresponds to subcritical flow conditions.

**Figure 15:**
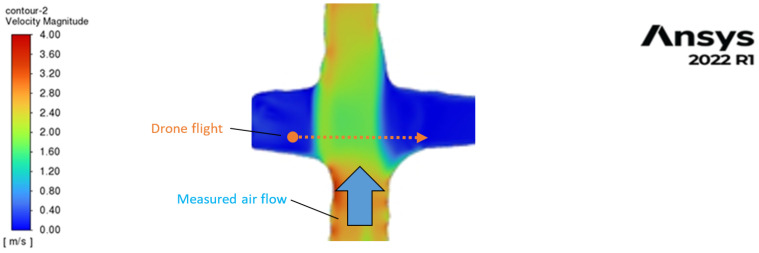
Demonstration by CFD simulation of airflow velocity measurement with the drone.

The demonstration by CFD simulation of the measurement method is represented in Figure 15, where the drone flies in one tunnel with weak flow (below 0.3 m/s) and measures the air velocity in another tunnel at the place of their crossing. In this case, the deviation of the drone trajectory will be to the left side since the air velocity will be higher on the right side.

Following CFD simulations and numerical calculations, we can conclude that the laboratory conditions of the experiments with drone flights in the corridor satisfy the three similarity criteria with a tunnel in the underground mine. Certainly, the unevenness of the tunnel surface will increase the Reynolds number, but it cannot change the type of flow (turbulent). The Froude number will be briefly affected by the deviations of the tunnel profile but can reach the critical value (Fr = 1) at flow velocities greater than 2.9–3.0 m/s. Hence, the proposed method can determine the average flow velocity at a certain altitude of the side tunnel outlet.

## 5. Discussion of Results

### 5.1. Measurement Data Processing

**Figure 16:**
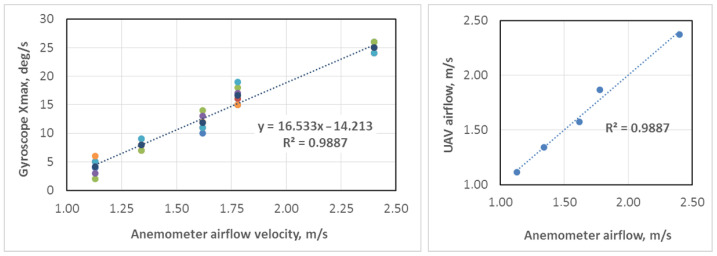
Comparison of airflow measurements with UAV and anemometer (coloured dots mean 1-5 experiments).

The mean values of six series of experiments for each of five values of air velocity are represented in Table 2, where the UAV values of the airflow are calculated using the linear approximation function from Figure 16. The maximum error of the proposed method is less than 5%, while the average error is about 2%. This precision is valid within the investigated range of airflow velocity (1.13–2.40 m/s). The wider range of airflow measurements will probably cause greater statistical deviations from the mean values, which require further investigation with more powerful airflows in laboratory experiments or calibration during field measurements in the mine.

The final formula for airflow calculation is as follows:

(6) $$V_{air}=k\cdot X_{max}-a,$$

where $V_{air}$—airflow velocity (m/s), $X_{max}$—maximum drone rotation speed around X (roll) axis (rad/s), *k*—coefficient of airflow in the drone flying tunnel (equal to 16.533 in our measurements), and *a*—bias of linear function (equal to −14.213 in our measurements). The extreme values of the drone rotation speed are calculated by the $max(X_{i})$ function if the disturbance acts from the left side (clockwise drone deviation) and the $min(X_{i})$ function if the disturbance is from the right side (counterclockwise deviation).

The maximum available measurement value of airflow velocity is estimated to be about 5 m/s with the UAV model used in the experiments. Generally, it depends on the size and power of the drone’s drives, but usually, used drones satisfy the regulations of underground mine ventilation inspection. In addition, this method allows for the airflow profile in the tunnel section to be determined.

### 5.2. Inspection Procedure

The procedure of the proposed method implementation in the practice of ventilation inspection is as follows:

- The UAV is launched by the operator at a certain point of the tunnel with a distance of 0.5 m from the ground, walls, and ceiling to prevent collisions with uneven rocks and turbulence. To keep a straight horizontal flight, the second member of an inspection team lights up by a laser pointer along the wall in the direction of further motion. This laser light should be 1.5–1.7 m from the floor, which usually corresponds to half of the tunnel’s vertical size in underground mines. In the case of higher tunnels, a special handle can be used to lift the laser pointer to a required altitude. The more convenient and precise flight control can be provided by the laser leveler, which shows the space limits for drone flight with vertical and horizontal planes.
- UAV is operated approximately at the altitude observed along the tunnel with a velocity greater than 2.0 m/s to reduce the disturbed air area. This value of horizontal velocity provides the deviation of the trajectory available for detection by small drones and minimal airflow (0.3 m/s) when the drone crosses the side tunnels. Small deviations from the altitude due to manual errors of the operator do not play a role in the measurements because they also exist in manual inspections.
- After crossing several side tunnels within the visible range, the UAV reverses its motion by the operator and flies back, repeating measurements. The flight distance depends on specific conditions and is usually about 40–50 m, within which several side tunnels are inspected.
- After the UAV returns to the operator, the segment of data recorded in the SD card is marked by the special drone motion maneuver (e.g., three jumps up and down) for better flight data separation during analysis.
- Then, the above-mentioned steps of procedure is repeated along the opposite wall to increase the accuracy of measurements. In the event of a suitable reliability of the airflow values obtained (checked with the anemometer for the first time), measurements can be conducted along the single wall without duplication on the opposite wall.
- If ventilation inspection regulation requires airflow measurements at different levels of the tunnel, the UAV is operated at the required heights, and the average values are calculated for every side tunnel.

The procedure for implementing the proposed method in the mining ventilation practice requires adaptation following existing inspection regulations.

Natural working conditions in underground mines with room and pillar exploitation systems create the basis for airflow velocity measurements by the UAV because of the absence of wind disturbances acting on the earth’s surface. That is why the inspection of ventilation systems can be scheduled at any suitable time without accounting for the weather prognosis.

In the UAV used in the experiments (DJI Mavic Mini), the optical stabilization system is based on vision data obtained from a vertically downward-oriented camera. When the test was conducted in the underground mine, this system was not feasible for existing ground patterns due to less reflection and less brightness of the surrounding light in the mine. Therefore, the influence of UAV’s stabilization system on trajectory deviations can be neglected in practice. In the case of more advanced UAV usage with other types of sensors and principles for stabilization, users should tune this function (if available in the settings) or use a simple drone without optical stabilization. This is an important advantage of the proposed method, which greatly reduces the costs of inspection instrumentation. However, in the case where there is no access to the flight log, an additional IMU module is required, which has an increased sampling frequency, allowing also a better resolution for the detection of UAV’s reaction on weak airflow.

By programming the UAV for the different altitudes of flight along the pillars, the user can measure airflow velocities at the corners or central points of the tunnel section, which are then averaged for ventilation productivity calculations.

## 6. Conclusions

The developed method of airflow velocity measurements is mostly suitable in underground mines with room and pillar exploitation systems when a network of long-distance crossed tunnels needs scheduled inspections of ventilation performance.

Using UAVs in ventilation systems inspection greatly reduces the time maintenance personnel spend in harsh environments (dust, high temperature, humidity, and exhaust gases).

The automatic procedure of data collection over long distances of underground tunnels (100–200 m for a single measurement and kilometers for the complete inspection mission) greatly improves the reliability of the results. In addition, digital data are provided for further analysis and modeling of ventilation systems.

The airflow velocity measurement accuracy achieved (maximum error of less than 5% and average error of about 2% compared with the anemometer) can be improved by further enhancing signal processing techniques, especially for the recognition of subsequent events in long data sets recorded during complicated UAV inspection missions.

Currently, only a linear flight route is assumed when disturbances from crossing perpendicular tunnels exceed all other factors of trajectory deviation. Including UAV maneuvers will result in more complicated gyroscope or acceleration signal processing procedures.

From the viewpoint of convenience and applicability, the new method is more efficient in the time required for one measurement and personnel health, but requires certain skills in drone operation with minimal qualification of computer users (data downloading and batch processing).

It should be noted that the positioning of DJI Mavic Mini drone utilized in this study, besides the GPS signal, depends on optical flow information, which provides limited stability, especially under poor lighting conditions. In our laboratory tests inside the building, the GPS signal was practically not available for UAV stabilization. This demonstrated the sufficiency of manual control with only visual drone stabilization for the proposed airflow measurement method. However, for real mining operations, additional positioning solutions and safety measures (propeller guards, obstacle avoidance sensors) are advised to ensure reliable and safe UAV performance. Commercially available UAVs specifically designed for GNSS-denied environments may offer more robust alternatives for deployment in underground conditions; however, they require special features in their flight control software.

## Figures and Tables

**Table 1:** Experiment results with anemometer axis inclination.

Air Velocity in Station (m/s)	Angle 0° (Normal)	Angle 10°	Angle 20°
0.50	0.30	0.30	0.30
3.40	2.80	2.80	2.78
5.60	5.54	5.56	5.52
8.80	7.38	7.34	7.26

**Table 2:** Results of airflow velocity measurements with UAV.

Anemometer (m/s)	Gyr. $X_{max}$ (deg/s)	St. Dev. (deg/s)	UAV (m/s)	Error (%)
1.13	4.2	1.2	1.11	1.62
1.34	8.0	0.7	1.34	0.27
1.62	11.8	1.2	1.58	2.75
1.78	16.7	1.3	1.87	4.93
2.40	25.0	0.7	2.37	1.17

## Data Availability

The measurement data presented in this study are not publicly available due to restrictions on privacy.
